# Latinx Individuals Who Smoke Daily with and without a Probable Anxiety Disorder: Differences in Smoking Behavior and Beliefs about Abstinence

**DOI:** 10.3390/ijerph20043277

**Published:** 2023-02-13

**Authors:** Michael J. Zvolensky, Bryce K. Clausen, Justin M. Shepherd, Brooke Y. Redmond, Lorra Garey, Luke F. Heggeness, Andre Bizier, Richard A. Brown, Daniel Bogiaizian, Patricio López Salazar

**Affiliations:** 1 Department of Psychology, University of Houston, Houston, TX 77004, USA; 2 Department of Behavioral Science, The University of Texas MD Anderson Cancer Center, Houston, TX 77030, USA; 3 HEALTH Institute, University of Houston, Houston, TX 77204, USA; 4 Health Behavior Solutions, Austin, TX 78702, USA; 5 Department of Psychology and School of Nursing, The University of Texas at Austin, Austin, TX 78712, USA; 6 Psychotherapeutic Area of “Asociación Ayuda”, Anxiety Disorders Clinic, Buenos Aires C1073AAO, Argentina; 7 Department of Psychology, Universidad Argentina de la Empresa, Buenos Aires C1073AAO, Argentina

**Keywords:** Latinx/Hispanic, health disparities, smoking, anxiety disorder, cigarette dependence, abstinence expectancies, perceived barriers for quitting

## Abstract

There is a well-established relation between anxiety psychopathology and smoking in the general population. However, little work focuses on Latinx/Hispanic (hereafter Latinx) persons who smoke from this comorbidity perspective. The present investigation aimed to explore differences among English-speaking Latinx adults who live in the United States (US) and smoke cigarettes with and without a probable anxiety disorder in terms of cigarette dependence, perceived barriers for quitting, severity of problems when quitting, and smoking abstinence expectancies. The sample included 338 adult Latinx daily cigarette smokers (*M_age_* = 35.53 years; *SD* = 8.65; age range 18–61; 37.3% female) who identified as Latinx and were recruited nationally throughout the US. Results indicated that among Latinx persons who smoke, those with a probable anxiety disorder (compared to those without) were more likely to demonstrate higher levels of cigarette dependence, severity of problems when trying to quit, perceived barriers for quitting, and negative abstinence expectancies after adjusting for key variables linked to smoking and anxiety (e.g., hazardous drinking, education). The current findings are the first to document probable anxiety disorder status as a clinically relevant factor for a wide range of smoking variables and beliefs about abstinence among Latinx persons who smoke.

## 1. Introduction

The Latinx/Hispanic (hereafter, Latinx) population in the United States (US) experiences unique and significant tobacco-related health disparities compared to non-Latinx smokers [1]. The prevalence of smoking is lower among Latinx persons when compared to non-Latinx White persons [2]. Further, the rate of smoking among Latinx persons who smoke is often lower than non-Latinx White individuals [2,3]. However, smoking is less likely to be detected and clinically addressed among Latinx persons relative to non-Latinx White individuals [4]. For example, Latinx persons who smoke are less likely to receive advice to quit, engage in smoking cessation treatment, or use evidence-based pharmacotherapy in efforts to quit cigarettes compared to non-Latinx White individuals [4,5,6]. Consequently, this group of Latinx smokers is apt to experience continued negative health consequences due to smoking, including many of the leading causes of death and disability for this population, e.g., cardiovascular disease, cancer, and diabetes [7].

Numerous factors influence smoking behavior, including genetic [8], environmental [9], and developmental [10] factors. There also has been increased recognition of the role of emotional states and disorders in the maintenance and relapse of smoking [11,12]. Among emotional phenotypes, anxiety states and disorders are among the most common in the general population and frequently co-occur with smoking [13], as smoking is often used as a mean to escape and/or avoid anxiety-related symptoms and sensations [14]. Indeed, drawing from largely non-Latinx White samples, smoking is more prevalent among those with elevated anxiety symptoms and anxiety psychopathology relative to the general population [11,15]. Across anxiety disorders, rates of smoking are highest among individuals with panic-related problems and other anxiety disorders where panic attacks are common [16,17,18,19,20,21,22,23]. Studies indicate anxiety disorders significantly impair successful smoking cessation and the maintenance of abstinence from smoking [24]. For example, one recent study found persons with anxiety disorders are less likely to stop smoking and more likely to smoke for longer periods of time [13]. Other research has found that treatment-seeking smokers with anxiety disorders compared to those without such disorders are more apt to experience poorer cessation outcomes [25]. Further, successful smoking cessation is associated with lower post-quit severity of anxiety symptoms [26].

Anxiety symptoms and disorders are among the most common forms of psychological distress among Latinx persons [27]. However, limited work has expressly focused on Latinx smokers in terms of anxiety symptoms and anxiety disorders. Within the available literature, there is evidence that greater anxiety symptoms, measured dimensionally, are associated with negative and positive outcome expectancies among Latinx Spanish-speaking smokers from the US [28] as well as greater perceived barriers for quitting and severity of interoceptive symptoms when trying to quit [29]. Related research has found that the cognitive factor of anxiety sensitivity, the belief that anxiety symptoms cause personal harm [30], is related to early smoking lapse among Latinx smokers from Mexico [31], negative and positive smoking outcome expectancies [32], as well as cigarette dependence and perceived barriers for quitting among Latinx Spanish-speaking smokers from the US [29,33]. Although anxiety symptoms and anxiety sensitivity are related, they are distinct constructs [34]. Nonetheless, the available ‘anxiety centric’ work on smoking among Latinx persons who smoke suggests that anxiety-related constructs are involved in several smoking processes.

Although work on smoking-anxiety relations among the Latinx population is growing, several limitations characterize its status. First, there has been no direct test as to whether having a probable anxiety disorder compared to not having a probable anxiety disorder is related to smoking behavior. This gap constrains empirical knowledge of anxiety psychopathology and the nature of smoking. To the extent anxiety disorders are involved in smoking, it would be expected that such clinical status should be associated with greater cigarette dependence [35], perceived barriers for quitting [36], and more severe symptoms when trying to quit [37]—clinically relevant smoking processes linked to the maintenance and relapse of smoking. On a practical level, this information may prove important when screening Latinx persons who smoke who may be at an increased risk for relapse and in need of specialized smoking cessation programming.

Second, past work also has not sought to examine the question of whether probable anxiety disorder status is related to beliefs about smoking abstinence. Abstinence expectancies pertain to the acute (i.e., immediate versus chronic) expected effects related to smoking abstinence [38]. There are both negative abstinence expectancies, including harmful consequences (e.g., “I would feel like I’m going crazy”), somatic symptoms (e.g., “My chest would feel tight”), and negative mood (e.g., “I would feel grouchy”), as well as positive consequence expectancies for abstinence e.g., “I would feel energized” [39]. Research among largely non-Latinx White individuals who smoke has found that negative abstinence expectancies are associated with numerous smoking behaviors (e.g., quit success) and other clinical correlates of tobacco use [40], e.g., cigarette dependence, motivation to quit, and severity of tobacco withdrawal [41]. To the extent that anxiety disorders bias threat-based thinking [42,43], in conjunction with the mood-modulating role of smoking [44], probable anxiety disorder status would be expected to be associated with negative abstinence expectancies among Latinx persons who smoke.

The present investigation aimed to explore differences among English-speaking Latinx adults living in the US who smoke, with and without a probable anxiety disorder, in terms of several clinically significant smoking processes. First, it was hypothesized that those with (compared to those without) a probable anxiety disorder would showcase greater cigarette dependence, severity of problems when quitting, and more perceived barriers for quitting. Second, it was hypothesized that clinical anxiety status would be related to heightened negative (negative mood, somatic symptoms, harmful consequences), but not positive smoking abstinence expectancies. These hypotheses were based upon past non-Latinx smoking research showcasing clinical anxiety-smoking relations [25] and the available limited literature suggesting continuous indices of anxiety are related to smoking processes among Latinx persons who smoke [28].

## 2. Materials and Methods

### 2.1. Participants

The current sample included 338 adult Latinx daily cigarette smokers (*M_age_* = 35.53 years; *SD* = 8.65; age range 18–61; 37.3% female). All participants identified as Hispanic/Latinx. In terms of race, participants identified as follows: 72.2% identified as Latinx White, followed by 10.9% other, 7.1% Latinx Black or African American, 4.1% Latinx Alaska Native or Latinx American Indian, 3.3% Multiracial/more than one race, 1.2% Latinx Native Hawaiian or other Latinx Pacific Islander, and 0.9% Latinx Asian. Of the participants, 76.0% reported working full time, 8.9% reported working part time, 2.4% were dependent as a spouse/student, and 12.4% were unemployed. Additionally, 39.6% reported an annual income of >$75,000, followed by 18.3% who reported an annual income between $50,000 and $74,999, 15.7% who reported an annual income between $35,000 and $49,000, with the remaining 26.4% who reported an annual income of $34,999 or lower. A total of 38.2% of participants indicated that their highest level of education was a bachelor’s degree, followed by 18.0% who completed a master’s degree, 13.3% who completed some college, 12.4% who completed high school or equivalent, 8.9% who completed an associate degree, 3.8% who completed some high school, 3.6% who obtained a doctoral degree, 1.4% who completed less than high school level, and 0.3% who obtained more than a doctorate level of education.

### 2.2. Procedures

All participants recruited for the current investigation were located nationally throughout the United States and were contacted using Qualtrics Panels, which is an online survey management system that has been implemented in past literature to gather reliable data for harder-to-reach populations [45,46]. To receive information about the study, all participants had to identify as Hispanic or Latinx, currently smoke combustible cigarettes daily, and own a Qualtrics Panels account. Participants who met these criteria were then sent an advertisement about the study, and those who expressed interest in participating completed a screening survey to determine eligibility. Following the screener, participants who were deemed eligible and were interested in participating were directed to an online anonymous survey. Eligible participants were at least 18 years old, self-reported smoking at least five cigarettes per day, and identified as Hispanic or Latinx. All participants were informed of the nature of the study and provided voluntary informed consent prior to completing the survey. Participants could opt to receive the equivalent of $10.75 in compensation for the study in the form of gift cards, rewards miles, or rewards points. Data safeguards were implemented to ensure data integrity and valid responses. These safeguards included recording IP addresses to prevent the same participant from completing the survey more than once and a speeding check (i.e., one-half the median survey completion time) to ensure participants were not answering randomly. This study was approved by the Institutional Review Board of the University where the study took place.

### 2.3. Measures

*Demographics Questionnaire.* All participants completed a demographics questionnaire. In the current study, age, sex (0 = Male, 1 = Female), highest level of education, and nativity (0 = born in the U.S., 1 = born in other country) were used as covariates.

*Alcohol Use Disorders Identification (AUDIT).* The AUDIT [47] is a 10-item self-report measure that assesses risky or hazardous drinking. Items (e.g., “How often during the last year have you needed a first drink in the morning to get yourself going after a heavy drinking session?”) are rated on a 5-point Likert scale. The AUDIT includes as total sore as well as three subscale scores (e.g., consumption). The hazardous drinking subscale in the AUDIT has been used in past research as a screener for problem drinking [48] and has been implemented successfully in past studies among Latinx smokers [28]. The 3-item hazardous drinking subscale was used as a covariate in the current investigation and demonstrated good internal consistency (α = 0.79).

*Drug Abuse Screening Test (DAST-10).* The DAST-10 [49] is a 10-item measure that assesses drug use problem severity. Individuals respond (0 = no, 1 = yes) to each item (e.g., “Are you always able to stop using drugs when you want to?”). Scores range from 0–10 with lower scores indicating no problems related to drug abuse and high scores indicating severe levels of drug abuse problems (0 = no problems reported, 1–2 = low levels, 3–5 = moderate levels, 6–8 = substantial levels, and 9–10 = severe levels). In the current investigation, the DAST-10 had good internal consistency (α = 0.86) and was used as a covariate.

*Fagerström Test for Cigarette Dependence-Revised*. The FTCD-R [50] is a 6-item scale that assesses degrees of cigarette dependence. Scores range from 0–16, with higher scores reflecting higher levels of physiological dependence on cigarettes. As in prior work, items 2, 5, and 6 were scored on a 4-point Likert-type scale ranging from 0 (*never*) to 3 (*always*). In the present study, the FTCD-R was used as a criterion variable and had low internal consistency (α = 0.64), but low internal consistency for the FTCD-R is common [50]. Past work indicates positive relations with biochemical markers of tobacco use (e.g., saliva cotinine), and high test–retest reliability [51,52], including among Latinx individuals who smoke [53].

*Barriers for Cessation (BCS).* The BCS [54] is a 19-item self-report measure of perceived barriers to or stressors resulting from smoking cessation (e.g., “Feeling less in control of your moods”). Responses are rated on a 4-point Likert scale ranging from 0 (*not a barrier*) to 3 (*large barrier*). The BCS has demonstrated strong psychometric properties in past work [36] and has been used among Latinx individuals who smoke in past work [55]. In the present study, the BCS total score demonstrated strong internal consistency (α = 0.95) and was utilized as a criterion variable.

*Smoking History Questionnaire (SHQ)*. The SHQ [56] measures smoking-related demographic information (e.g., age of regular daily smoking and total number of years smoking daily, number of failed quit attempts) and is also used to assess the severity of problems experienced during prior quit attempts. The SHQ was used to assess years of daily smoking (“For how many years, altogether, have you been a regular daily smoker?”), number of past quit attempts (“How many times in your life have you made a serious attempt to quit smoking?”), and severity of problems experienced during quit attempts [56]. As in past work [37], a mean composite score of severity of problem symptoms experienced during past quit attempts was derived from this measure. Specifically, this item asks, “While trying to quit, how serious have each of the following problems been for you?” Examples of the 17 items include weight gain, headaches, irritability, or insomnia. Items were rated on a 5-point Likert-type scale from 1 (*not at all*) to 5 (*extremely*). The severity of these items was averaged to create a composite score and utilized as a criterion variable. The composite score demonstrated strong internal consistency (α = 0.96), as in past smoking research [33,57]. In addition, the average number of cigarettes smoked per day was used as a covariate.

*Smoking Abstinence Expectancies Questionnaire (SAEQ).* The SAEQ [39] measures participants expected acute consequences resulting from smoking abstinence via 28-items. Each item is rated on a 7-point Likert scale ranging from 0 (*very unlikely*) to 6 (*very likely*). The SAEQ has four subscales, with higher scores representing stronger beliefs about abstinence expectancies. Three subscales represent negative abstinence expectancies, including negative mood (e.g., “I would have trouble getting along with difficult people”), somatic symptoms (e.g., “My throat would feel dry”), and harmful consequences (e.g., “I would have a panic attack.”). A fourth subscale reflects positive expectancies (positive consequences, e.g., “I would find it easy to concentrate”). The SAEQ has demonstrated, in past research, good psychometric properties including test-retest reliability, good internal consistency, and convergent and discriminant validity [39,58,59]. The four subscales were utilized in the current study as dependent variables, and all demonstrated excellent internal consistency (α’s range = 0.90–0.91).

*Overall Anxiety Severity and Impairment Scale (OASIS).* The OASIS [60] measures anxiety and level of impairment due to anxiety through 5 items. Participants are instructed to rate each item (e.g., “In the past week, how often did you avoid situations, places, objects, or activities because of anxiety or fear?”) on a scale ranging from 0–4 with anchors specific to each item (e.g., 0 = *none*; 4 = *all the time*). In the current investigation, the OASIS demonstrated excellent internal consistency (α = 0.94) and was used as a group variable for probable anxiety. Past research has indicated that a cutoff score of ≥8 reflects a probable anxiety disorder [61].

### 2.4. Analytic Strategy

A post-hoc power analysis was conducted. Analyses were conducted using SPSS version 28. First, descriptive statistics were computed (see Table 1). Second, seven independent analysis of covariance (ANCOVA) models were conducted to evaluate differences in cigarette dependence, barriers for smoking cessation, problems experienced during prior quit attempts, and the four smoking abstinence expectancy subscales, including negative mood, somatic symptoms, harmful consequences, and positive consequences for Latinx adult smokers with and without a probable anxiety disorder. Interpretations of effect sizes were categorized using the following values: small = 0.01, medium = 0.06, and large = 0.14 [62]. An alpha correction was used (0.05/7 = 0.007) for the number of comparisons. Covariates for all analyses included age [63,64], sex [41,65], education [66], nativity [33], number of cigarettes smoked per day [67], hazardous drinking [68], and drug abuse [69,70,71]. Partial eta squared (η_p_^2^) was used as an index of effect size [72].

## 3. Results

Power analysis indicated a sample size of 338 was sufficient to detect medium effects. Descriptive statistics are presented in Table 1; 50.9% of the sample met the cutoff score (≥8) indicating a probable anxiety disorder [61].

See Table 2 for group differences. For cigarette dependence, controlling for covariates, a statistically significant difference emerged between probable anxiety disorder versus no probable anxiety disorder [*F* (1, 338) = 8.51, *p* = 0.004]; a small effect size was observed (η_p_^2^ = 0.025). Adjusted mean differences were *M* = 7.13 (*SE* = 0.21) for those with a probable anxiety disorder and *M* = 6.21 (*SE* = 0.21) for those with no probable anxiety disorder.

For barriers for cessation, controlling for covariates, a statistically significant difference emerged between probable anxiety disorder versus no probable anxiety disorder [*F* (1, 338) = 45.03, *p* ≤ 0.001]; a medium effect size was observed (η_p_^2^ = 0.120). Adjusted mean differences were *M* = 33.03 (*SE* = 0.93) for those with a probable anxiety disorder and *M* = 23.70 (*SE* = 0.94) for those with no probable anxiety disorder.

For severity of quit problems, controlling for covariates, a statistically significant difference emerged between probable anxiety disorder versus no probable anxiety disorder [*F* (1, 338) = 38.52, *p* ≤ 0.001]; a medium effect size was observed (η_p_^2^ = 0.105). Adjusted mean differences were *M* = 3.23 (*SE* = 0.07) for those with a probable anxiety disorder and *M* = 2.57 (*SE* = 0.07) for those with no probable anxiety disorder.

For negative mood abstinence expectancies, controlling for covariates, a statistically significant difference emerged between probable anxiety disorder versus no probable anxiety disorder [*F* (1, 338) = 19.02, *p* ≤ 0.001]; a medium effect size was evident (η_p_^2^ = 0.055). Adjusted mean differences were *M* = 26.96 (*SE* = 0.72) for those with a probable anxiety disorder and *M* = 22.24 (*SE* = 0.74) for those with no probable anxiety disorder.

For somatic symptom abstinence expectancies, controlling for covariates, a statistically significant difference emerged between probable anxiety disorder and no probable anxiety disorder [*F* (1, 338) = 10.53, *p* ≤ 0.001]. The effect was small (η_p_^2^ = 0.031). Adjusted mean differences were *M* = 23.80 (*SE* = 0.73) for those with a probable anxiety disorder and *M* = 20.25 (*SE* = 0.74) for those with no probable anxiety disorder.

For harmful-consequence abstinence expectancies, controlling for covariates, a statistically significant difference emerged between probable anxiety disorder and no probable anxiety disorder [*F* (1, 338) = 18.91, *p* ≤ 0.001]. The effect was small (η_p_^2^ = 0.054). Adjusted mean differences were *M* = 24.25 (*SE* = 0.73) for those with a probable anxiety disorder and *M* = 19.52 (*SE* = 0.74) for those with no probable anxiety disorder.

For positive consequences abstinence expectancies, controlling for covariates, no statistically significant difference emerged between probable anxiety disorder and no probable anxiety disorder [*F* (1, 338) = 5.37, *p* = 0.021]. Adjusted mean differences were *M* = 18.32 (*SE* = 0.73) for those with a probable anxiety disorder and *M* = 20.87 (*SE* = 0.75) for those with no probable anxiety disorder.

## 4. Discussion

Although there is a well-established relationship between anxiety psychopathology and smoking in the general population [12], there has been little work focused on racial/ethnic minorities from this comorbidity perspective. Such a limitation is unfortunate, as the presence of clinical anxiety among smokers has been associated with more severe smoking behavior [73], greater risk for relapse [25], and the need for more intensive or specialized treatment [74]. The current work sought to extend past research by exploring differences in smoking behavior and beliefs among Latinx adults who smoke with and without a probable anxiety disorder.

As hypothesized, Latinx persons who smoke with a probable anxiety disorder, compared to those without, were more likely to demonstrate higher levels of cigarette dependence, severity of problems when trying to quit, and greater perceived barriers for quitting. These significant effects were small to medium in magnitude, but evident above and beyond the variance associated with a range of theoretically relevant covariates (e.g., age, education, degree of hazardous drinking). Thus, these small to medium effects may be clinically significant [75]. Such findings extend past work on Latinx persons who smoke [28] by showcasing consistent group differences for those experiencing anxiety psychopathology versus those that do not in terms of a range of clinically significant processes linked to the maintenance and relapse of smoking [25].

Furthermore, as hypothesized, there were significant anxiety-based group differences in terms of smoking abstinence expectancies. Specifically, Latinx persons who smoke with a probable anxiety disorder compared to those without a probable anxiety disorder demonstrated heightened beliefs that smoking abstinence (acutely) would be associated with negative mood, somatic perturbation, and harmful consequences; the effect sizes were small. No differences were evident for positive abstinence expectancies, suggesting clinical anxiety status is more likely to be linked to ‘negative’ abstinence expectancies. These findings are in line with theoretical models of smoking–anxiety interplay [76] and related research among Latinx smoking samples [32] that suggests anxiety-related states may be involved in biased smoking beliefs centered on mood and bodily regulation. The group differences for clinical anxiety (versus not) are likely to be clinically important given abstinence expectancies are often related to less quit success and higher degrees of tobacco withdrawal and craving [39,77].

The current data suggest, in conjunction with non-Latinx smoking research [25], that it is likely to be clinically important to screen for probable anxiety disorder among Latinx smoking populations to better address the unique needs of this group. Indeed, Latinx individuals who smoke with a probable versus without a probable anxiety disorder showcased smoking-related risk across a broad band of indicators. Thus, this group is likely at a higher risk for smoking maintenance and relapse, perpetuating health risks linked to this addictive behavior in this group [3]. Moreover, the data suggest the need for a connection between assessment and treatment that addresses clinical anxiety in the context of smoking cessation. In largely non-Latinx White samples of individuals who smoke, there has been evidence from randomized controlled clinical trials showing that reducing the severity of anxiety symptoms or sensitivity to such symptoms using integrative care methods improves smoking cessation and mental health relative to standardized care [74,78,79,80]. Uncontrolled research with such integrated anxiety-smoking protocols focused on Latinx persons who smoke have also been promising [81]. However, there has been no effort to test the efficacy of such treatments for the Latinx smoking population using controlled research designs. This type of work, especially if further culturally adapted to Latinx smokers [82], may be an important next step in public health efforts to reduce the physical and mental health burden related to tobacco among this group.

There are several study limitations. First, we used established probable anxiety disorder cutoffs on the OASIS [61]. Although the OASIS is a validated instrument with strong psychometric properties [61], we did not employ a structured clinical interview that would permit a more granular understanding of clinical anxiety in terms of the studied smoking processes. For example, it may be useful to understand what type of anxiety disorder confers the greatest risk for smoking, or if having multiple anxiety conditions heightens smoking vulnerability relative to having few conditions. Past work among largely non-Latinx smoking samples indicates that there is heterogeneity in anxiety phenotype and extent of comorbidity for smoking behavior [15]. Future research employing structured clinical interviews of anxiety disorders among Latinx persons who smoke is therefore needed. Second, the cross-section research design does not permit causal-oriented hypothesis testing. Thus, the directionality of the observed associations cannot be fully explicated. Future research that examines clinical anxiety among Latinx individuals who smoke using controlled methodologies would be a useful research step. For instance, testing if anxiety psychopathology (versus not) is related to real-time expression of tobacco withdrawal, craving, and smoking topography in the laboratory or using ecological momentary assessment among the Latinx smoking population would provide important information about interrelations anxiety-smoking. Third, we sampled English-speaking Latinx individuals who smoke and were recruited through a Qualtrics Panel. Future research could explore the present model among a community-recruited sample of monolingual and polylingual Latinx Spanish-speaking and English-speaking individuals who smoke to better test the generalizability of the findings. Finally, the present sample was not treatment-seeking for smoking cessation. Therefore, there is a need for future research to explore the role of clinical anxiety among Latinx persons who smoke and are engaged in smoking cessation to better understand how anxiety psychopathology is related to treatment engagement (e.g., attendance) and outcome (e.g., quit success). Previous smoking cessation research among the Latinx population suggests that mental health issues among this population are important processes related to smoking relapse [83].

## 5. Conclusions

Overall, the present work adds to a small but growing clinical literature [29] that demonstrates the importance of clinical anxiety in terms of smoking behavior and beliefs among Latinx persons who smoke. Across a diverse range of clinically significant smoking variables, those individuals with versus those without a probable anxiety disorder had greater smoking problems and endorsed more negative beliefs about smoking abstinence. Future work is needed to understand the role of clinical anxiety in smoking relapse in the Latinx population, which is an established tobacco disparities population [1].

## Figures and Tables

**Table 1:** Descriptive statistics.

Variable	Mean (SD)/N [%]
Age	35.35 (8.65)
Biological Sex	126 [37.3%]
Nativity	43 [12.7%]
Average Number of Cigarettes Per Day	10.78 (8.77)
Hazardous Drinking	4.88 (8.77)
Drug Abuse	2.98 (2.90)
Probable Anxiety	172 [50.9%]
Cigarette Dependence	6.68 (3.02)
Barriers to Cessation	28.45 (14.57)
Quit Problems	2.90 (1.07)
Negative Mood	24.64 (9.81)
Somatic Symptoms	22.06 (10.27)
Harmful Consequences	21.93 (10.34)
Positive Consequences	19.57 (10.16)

*Note.* N = 338, Biological Sex = % female; Nativity % shown for those born outside the US (Coded as 0 = Born in the US, 1 = Not born in the US); Hazardous Drinking = AUDIT Hazardous Drinking Subscale [47]; Drug Abuse Problems = DAST-10 Total Score [49]; Probable Anxiety = OASIS Total Score [60]; Cigarette Dependence = FTCD Total Score [50]; Barriers to Cessation = BCS Total Score [54]; Quit Problems = SHQ Quit Problems Subscale [56]; Negative mood = SAEQ negative mood subscale [39]; Somatic Symptoms = SAEQ somatic symptoms subscale [39]; Harmful Consequences = SAEQ harmful consequences subscale [39]; Positive Consequences = SAEQ positive consequences subscale [39].

**Table 2:** *ANCOVA*.

**Cigarette Dependence**	**SS**	**DF**	**MS**	**F**	**Sig.**	**η_p_^2^**
Age	1.46	1	1.46	0.22	0.643	0.001
Sex	4.30	1	4.30	0.63	0.427	0.002
Education	25.98	1	25.98	3.83	0.051	0.011
Nativity	15.40	1	15.40	2.27	0.133	0.007
Cigarettes Per Day	205.91	1	205.91	30.33	<0.001	0.084
Hazardous Drinking	33.82	1	33.82	4.98	0.026	0.015
Drug Abuse	146.33	1	146.33	21.55	<0.001	0.061
Probable Anxiety	57.78	1	57.78	8.51	0.004	0.025
Total	1814	338				
**Barriers to Cessation**	**SS**	**DF**	**MS**	**F**	**Sig.**	**η_p_^2^**
Age	623.25	1	623.25	4.71	0.031	0.014
Sex	70.55	1	70.55	0.53	0.466	0.002
Education	2214.02	1	2214.02	16.73	<0.001	0.048
Nativity	294.44	1	294.44	2.23	0.137	0.007
Cigarettes Per Day	14.95	1	14.95	0.11	0.737	<0.001
Hazardous Drinking	1094.29	1	1094.29	8.27	0.004	0.025
Drug Abuse	3445.40	1	3445.40	26.04	<0.001	0.073
Probable Anxiety	5958.56	1	5958.56	45.03	<0.001	0.120
Total	345,108	338				
**Quit Problems**	**SS**	**DF**	**MS**	**F**	**Sig.**	**η_p_^2^**
Age	4.91	1	4.91	6.30	0.013	0.019
Sex	0.27	1	0.27	0.35	0.555	0.001
Education	7.72	1	7.72	9.91	0.002	0.029
Nativity	1.67	1	1.67	2.14	0.144	0.006
Cigarettes Per Day	2.58	1	2.58	3.32	0.07	0.010
Hazardous Drinking	4.27	1	4.27	5.48	0.02	0.016
Drug Abuse	13.63	1	13.63	17.51	<0.001	0.051
Probable Anxiety	30.00	1	30.00	38.52	<0.001	0.105
Total	3234.172	338				
**Negative Mood**	**SS**	**DF**	**MS**	**F**	**Sig.**	**η_p_^2^**
Age	67.30	1	67.30	0.84	0.36	0.003
Sex	12.98	1	12.98	0.16	0.687	<0.001
Education	296.25	1	296.25	3.70	0.055	0.011
Nativity	266.92	1	266.92	3.33	0.069	0.010
Cigarettes Per Day	81.24	1	81.24	1.02	0.314	0.003
Hazardous Drinking	56.15	1	56.15	0.70	0.403	0.002
Drug Abuse	940.57	1	940.57	11.75	<0.001	0.034
Probable Anxiety	1522.46	1	1522.46	19.02	<0.001	0.055
Total	237,669.0	338				
**Somatic Symptoms**	**SS**	**DF**	**MS**	**F**	**Sig.**	**η_p_^2^**
Age	185.56	1	185.56	2.27	0.133	0.007
Sex	93.63	1	93.63	1.15	0.285	0.003
Education	866.48	1	866.48	10.60	0.001	0.031
Nativity	15.89	1	15.89	0.19	0.66	0.001
Cigarettes Per Day	55.32	1	55.32	0.68	0.411	0.002
Hazardous Drinking	574.25	1	574.25	7.02	0.008	0.021
Drug Abuse	1264.47	1	1264.47	15.47	<0.001	0.045
Probable Anxiety	861.03	1	861.03	10.53	0.001	0.031
Total	199,967.0	338				
**Harmful Consequences**	**SS**	**DF**	**MS**	**F**	**Sig.**	**η_p_^2^**
Age	122.60	1	122.60	1.51	0.22	0.005
Sex	2.44	1	2.44	0.03	0.862	<0.001
Education	624.38	1	624.38	7.70	0.006	0.023
Nativity	8.37	1	8.37	0.10	0.748	<0.001
Cigarettes Per Day	94.82	1	94.82	1.17	0.28	0.004
Hazardous Drinking	357.46	1	357.46	4.41	0.037	0.013
Drug Abuse	1675.58	1	1675.58	20.67	<0.001	0.059
Probable Anxiety	1532.99	1	1532.99	18.91	<0.001	0.054
Total	198,539.0	338				
**Positive Consequences**	**SS**	**DF**	**MS**	**F**	**Sig.**	**η_p_^2^**
Age	28.55	1	28.55	0.35	0.557	0.001
Sex	14.15	1	14.15	0.17	0.68	0.001
Education	827.85	1	827.85	10.0	0.002	0.029
Nativity	54.78	1	54.78	0.66	0.417	0.002
Cigarettes Per Day	0.17	1	0.17	0.002	0.964	<0.001
Hazardous Drinking	979.08	1	979.08	11.82	<0.001	0.035
Drug Abuse	1067.14	1	1067.14	12.88	<0.001	0.038
Probable Anxiety	444.89,	1	444.89	5.37	0.021	0.016
Total	198,539	338				

*Note.* SS = Sum of Squares; MS = Mean Square; Sex (Coded: 1 = male, 2 = Female); Education (Coded: 1 = Less than high school, 2 = Some high school, 3 = Completed high school (or equivalent), 4 = Some college, 5 = Associate’s Degree, 6 = Bachelor’s Degree, 7 = Master’s Degree, 8 = Doctoral Degree, 9 = More than Doctorate); Nativity (Coded as 0 = Born in the US, 1 = Not born in the US); Hazardous Drinking = AUDIT Hazardous Drinking Subscale [47]; Drug Abuse Problems = DAST-10 Total Score [49]; Probable Anxiety = OASIS Total Score ≥ 8 [60]; Cigarette Dependence = FTCD-R Total Score [50]; Barriers to Cessation = Barriers to Cessation Scale [54]; Quit Problems = SHQ total score [56]; Negative mood = SAEQ negative mood subscale [39]; Somatic Symptoms = SAEQ somatic symptoms subscale [39]; Harmful Consequences = SAEQ harmful consequences subscale [39]; Positive Consequences = SAEQ positive consequences subscale [39].

## Data Availability

Data available on request due to restrictions of privacy.
